# Dendritic Cell-Mediated Th2 Immunity and Immune Disorders

**DOI:** 10.3390/ijms20092159

**Published:** 2019-05-01

**Authors:** Sunil Kumar, Yideul Jeong, Muhammad Umer Ashraf, Yong-Soo Bae

**Affiliations:** 1 Science Research Center (SRC) for Immune Research on Non-Lymphoid Organ (CIRNO), Sungkyunkwan University, Jangan-gu, Suwon, Gyeonggi-do 16419, Korea; sunilkumar@skku.edu (S.K.); skyiller@gmail.com (Y.J.); drumerashraf@gmail.com (M.U.A.); 2 Department of Biological Science, Sungkyunkwan University, Jangan-gu, Suwon, Gyeonggi-do 16419, Korea

**Keywords:** dendritic cells, Th2 immunity, genetic factors, environmental factors, Th2 disorders, therapeutic approaches

## Abstract

Dendritic cells (DCs) are the professional antigen-presenting cells that recognize and present antigens to naïve T cells to induce antigen-specific adaptive immunity. Among the T-cell subsets, T helper type 2 (Th2) cells produce the humoral immune responses required for protection against helminthic disease by activating B cells. DCs induce a Th2 immune response at a certain immune environment. Basophil, eosinophil, mast cells, and type 2 innate lymphoid cells also induce Th2 immunity. However, in the case of DCs, controversy remains regarding which subsets of DCs induce Th2 immunity, which genes in DCs are directly or indirectly involved in inducing Th2 immunity, and the detailed mechanisms underlying induction, regulation, or maintenance of the DC-mediated Th2 immunity against allergic environments and parasite infection. A recent study has shown that a genetic defect in DCs causes an enhanced Th2 immunity leading to severe atopic dermatitis. We summarize the Th2 immune-inducing DC subsets, the genetic and environmental factors involved in DC-mediated Th2 immunity, and current therapeutic approaches for Th2-mediated immune disorders. This review is to provide an improved understanding of DC-mediated Th2 immunity and Th1/Th2 immune balancing, leading to control over their adverse consequences.

## 1. Introduction

Dendritic cells (DCs) are the professional antigen-presenting cells (APCs) that play an important role in immune defense by activating the adaptive immune system. DCs were first discovered by Steinman and Cohn in 1973 [1], and extensive studies have since been conducted related to the various DC subsets in humans and mice and their characteristics [2]. Different DC subsets or the same DC subset in different environments can induce different T-cell immunity [3,4,5]. T helper type 2 (Th2) immunity mainly performs two important interconnected functions, i.e., providing direct protection against the extracellular parasites (helminths), but this protective immunity occasionally leads to adverse reactions, such as an allergic response [6,7,8]. The protective functions of Th2 immunity for pathogen clearance are mediated by the induction of Th2 cytokines (interleukin 4 (IL-4), 5 (IL-5), 6 (IL-6), 10 (IL-10), and 13 (IL-13)) and the recruitment of B cells and eosinophils, while allergic responses are mediated by hypersecretion of IgE from B cells and histamines from mast cells and basophils. Genetic and environmental factors have also been considered to influence the Th2 immune response. Environmental factors include pathogens (bacteria, viruses, fungi, and parasites), foods, and allergens (house dust mites (HDMs), pollens, etc.), whereas genetic factors include the specific genes of DCs essential for induction of Th2 immunity. Other factors, such as hormones and lipids, also affect Th2 immunity, but details on these subjects are beyond the scope of this review. This review mainly covers DC-mediated Th2 immunity.

**Figure 1:**
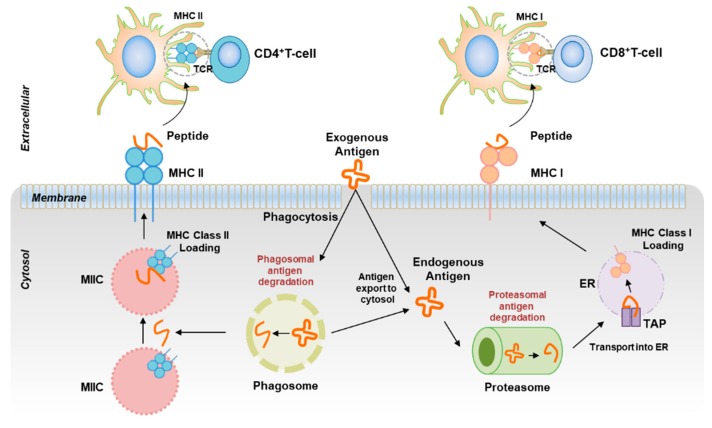
Schematic diagram for the cross-presentation machinery of DCs. DCs take up extracellular antigen, process it to antigenic peptides, and present these peptides not only with MHC II through the MIIC to CD4^+^ T cells (**left**), but also with MHC I through proteasome and the TAP/ER pathway to CD8^+^ T cells (**right**).

DCs are the first lines of immune defense that come into play once encountered with a foreign antigen and decide whether to tolerate or elicit a strong immune response against it. If the response has already intensified, the immune system must decide what kind of response is appropriate to clear the pathogens. To accomplish this task, DCs engulf and present these antigens via cross-presentation machinery to CD4^+^ naïve T or CD8^+^ T cells (Figure 1), leading to the birth of a specific T-cell subset, like T helper types 1 (Th1), 2 (Th2), and 17 (Th17), and T regulatory (Treg) subsets or cytotoxic T lymphocytes (CTLs) to orchestrate immune responses in both humans and mice [9]. A pool of cytokines released during this process actually governs the fates of these T cells to acquire specific T-cell polarity. Differentiation of Th1 cells is triggered by interleukin 12 (IL-12) and characterized by high-level secretion of cytokines: Interferon gamma (IFN-γ), interleukin 2 (IL-2), and lymphotoxin, which recruits macrophages, B-lymphocytes, CD8^+^ T cells, natural killer (NK) cells, and neutrophils to the site of infection to impart protective Th1 immunity in both humans and mice [10]. Conversely, Th2 cells are triggered by IL-4 and characterized by high-level secretion of IL-4, IL-5, and IL-13, which activate B cells to produce immunoglobulin E (IgE) and recruit basophils to mediate Th2-specific immune responses in both humans and mice [11]. Despite the crucial role of T-cell subsets in host defense, they are also associated with severe immune pathologies, including hypersensitivity, tumorigenesis, and autoimmunity [12]. The mechanisms controlling the differentiation of Th1, Th17, and Treg cells are well described. However, the details of Th2 differentiation by specific subsets of DCs remain controversial. Gao et al. (2013) investigated the role of the IRF4^+^ DC subset in initiating the Th2 response in mice [13]. Murphy et al. (2015) suggested that a Klf4-expressing DC subset is required for Th2 responses in mice [14]. A recent study by Ahmed et al. (2017) has shown that a DC subset expressing SH2 domain-containing adaptor protein B (SHB) ensures Th2 homeostasis by regulating DC-mediated Th2 immunity in an atopic dermatitis (AD) mouse model [15]. These findings prompted us to investigate the role of these genes in DC-mediated Th2 immunity. In this review, we discuss DC subsets and genes of interest in DCs essential for induction of Th2 immune responses in the context of allergic diseases and illustrate the well-coordinated interplay of different signaling pathways, such as transcription factors, cytokines, epigenetics, and microRNAs, which are essential to obtain an optimal Th2 response.

## 2. T Cell Immunity

### 2.1. T Cell Development from Naïve T Cells

**Figure 2:**
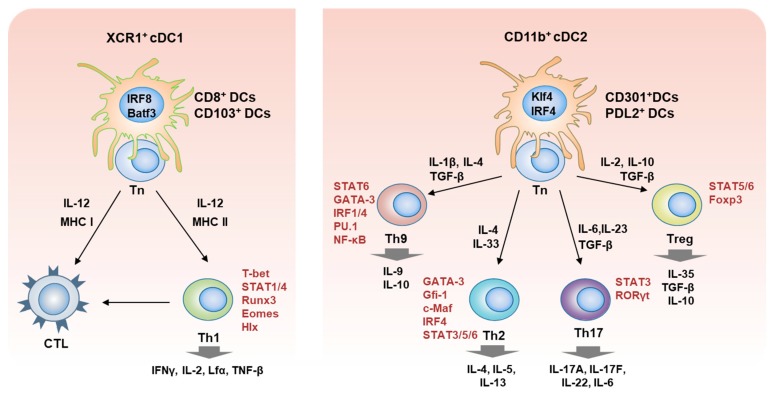
Schematic diagram for the involvement of cDC1 and cDC2 subsets in the T cell development. The XCR1^+^ cDC1, having major transcription factors, IRF8 and Batf3, are involved in the differentiation of naïve T cells into cytotoxic T lymphocytes (CTL) and Th1 cells with the cDC1 cytokine, IL-12 (**left**). Whereas, CD11b^+^ cDC2 known by their IRF4 and Klf4 facilitate the T cell development into Th2, Th9, Th17, and Treg cells with cDC2 cytokines (**right**). Key transcription factors mediating the development of each T cell subset are indicated in red. Each T cell subset secretes the effector cytokines as described.

The two major subsets of T lymphocytes, CD4^+^ T helper (Th) and CD8^+^ cytotoxic T lymphocytes (CTL), are distinguished by specific cell surface markers. DCs have the ability to differentiate naïve CD4^+^ T cells into different Th subsets, which requires three important signals (signal 1, 2, and 3) [16]. The signal 1 initiates the primary binding of APCs to antigen-specific T cells through interaction between antigenic peptide-loaded MHC II (class II major histocompatibility complex) and T-cell receptor (TCR) to induce the antigen-specific T cell response. The signal 2 is involved in T cell stimulation via interaction between the B7 costimulatory molecules on the DCs and CD28 on T cells. The third signal constitutes polarizing cytokines crucial for CD4^+^ T-cell differentiation into functional effector T-cell subsets. The signals obtained from the polarizing cytokines lead to the expression of specific transcription factors that direct the expression of effector cytokines and thus specialized Th-cell subsets, such as T-bet^+^IFN-γ^+^ Th1, GATA-3^+^IL-4^+^ Th2, STAT6^+^IL-9^+^ Th9, STAT3^+^Rorγt^+^IL-17^+^ Th17, and Foxp3^+^STAT5^+^CD25^+^ Treg cells (Figure 2) [10,17]. Th9 cells play a role in defense against helminth infections, in allergic responses, in autoimmunity, and tumor suppression [18], but their functions are still unclear. Th17 cells are involved in maintaining mucosal barriers and contributing to pathogen clearance at mucosal surfaces, but they have also been implicated in autoimmune and inflammatory disorders [19,20]. Treg cells modulate the immune system by suppressing or downregulating induction and proliferation of effector T cells, thus leading to maintenance of self-tolerance [21] and prevention of autoimmune disease [22]. Among these Th subsets, we expand this review to the Th1 and Th2, and finally more to the Th2 immunity.

### 2.2. Th1 and Th2 Responses

Each T-cell subset originating from naïve T cells takes charge of a specialized function and is recognized by specific characteristics. Th1 cells are characterized by high-level expression of IFN-γ, IL-2, lymphotoxin α, and tumor necrosis factor-beta (TNF-β) in response to IL-12 signaling. Th1 cells induced against intracellular parasites, such as protozoa, bacteria, viruses, and fungi, which recruit macrophages, neutrophils, NK cells, cytotoxic T-cells, B cells, and microglial-like effector cells to eliminate invaders by activating cell-mediated immune responses. Over-activation of Th1 cells leads to organ-specific autoimmune diseases, such as hypersensitivity, arthritis, and type 1 diabetes. The major transcription factors initiating Th1 cell differentiation are T-bet, STAT4, STAT1, Runx 3, Eomes, Hlx, etc. [12]. Th2 cells, in contrast, are characterized by expression of IL-4, IL-5, and IL-13 in response to IL-4 signaling. Th2 cells induced against extracellular parasites, mainly helminths and allergens, recruit B cells to produce IgE antibodies, basophils, eosinophils, and mast cells to eliminate the parasites by activating humoral and cell-mediated immune responses. Over-activation of Th2 immunity can lead to systemic autoimmune inflammatory diseases, such as allergies and AD. The major transcription factors initiating Th2 cell differentiation are GATA3, STAT6, STAT5, STAT3, Gfi-1, c-Maf, and IRF4 [23], as summarized in Figure 2.

### 2.3. Special Characteristics of Th2 Response

IL-4 is the key cytokine in Th2 immunity, but for many years, immunologists faced a “Th2 paradox.” Th2 development from naïve T cells requires activation of signal transducer and activator of transcription 6 (STAT6) downstream of the IL-4 receptor-signaling pathway, but the only known source of IL-4 was the Th2 cell itself. Today, however, other cell types, such as basophils [24,25], mast cells [26,27], and NKT cells [28], are known to serve IL-4 sources in both humans and mice. Among the transcription factors involved in developing T-cell subsets, GATA3 [29] and STAT6 [30] are the most important for Th2 differentiation from naïve CD4^+^ T cells in both humans and mice. Two major signaling pathways, IL-2/STAT5 and IL-4/STAT6, play a crucial role in Th2 differentiation. IL-2/STAT5 signaling implies that IL-2 receptor signaling initiates STAT5 activation, leading to the expression of IL-4 [12]. In the following IL-4/STAT6 pathway, IL-4 receptor signaling entails phosphorylation of STAT6 monomers, resulting in dimerization and translocation into the nucleus. In the nucleus, STAT6 dimers activate GATA3, which binds to the promoters of IL-4, IL-5, and IL-13, leading to the expression of these Th2 driving cytokines in both humans and mice [31].

## 3. Immune Cells Other than DCs and Cytokines in Polarizing Th2 Immunity

### 3.1. Basophils and Mast Cells

Basophils and mast cells are the major source of IL-4 inducers in mediating Th2 immunity. Basophils comprise 0.5% to 1% of total blood cells categorized as granulocytes. They contain inflammatory cytokines, such as histamines and heparins, which are known to cause harmful allergic reactions, but also help eliminate parasitic infections. Nakanishi et al. (2010) [32,33] proposed that basophils act as APCs and trigger Th2 immune responses through three pathways: First, by secreting IL-4 upon binding of allergen-IgE to its FcεRI surface receptor; second, through IL-3- and IL-33-dependent stimulation of basophils to secrete IL-4; and third, through pathogen-associated molecular pattern (PAMP) and toll-like receptor (TLR) ligand-associated direct stimulation of basophils in secreting IL-4. Another study with CD11c- DTR (diphtheria toxin receptor) mice showed that DC alone is not sufficient to induce a Th2 immune response, but it requires cooperation with basophils to enhance IL-4 production in response to protease allergen papain [34,35,36]. Collectively, these findings suggest that basophils behave as an accessory cell to support DCs in inducing Th2 immune response by serving IL-4 cytokines.

### 3.2. Innate Lymphoid Cells (ILCs)

ILCs, which consist of three subsets derived from the same progenitor cells, were recently discovered to be involved in initiating T-cell responses. Among the three groups, only the ILC2 group was found to be involved in Th2 immune responses in response to IL-4, IL-25, and IL-33 [37,38]. It activates in response to parasites and allergens and contributes to allergic inflammatory diseases, such as asthma, chronic rhinitis, and AD [3]. Recently Kim et al. (2013) found that ILC2 cells are present on healthy skin as well as lesional skin, which contributes to inflammatory responses in AD. The ILC2 cells on AD lesion skin are characterized by high-level expression of CD25/IL-33R/CRTH2/CD161, which are found at low levels in healthy skin [39].

### 3.3. Epithelial Cells (ECs)

ECs in the outer barrier of our body constantly encounter invasive or inhaled pathogens and allergens, resulting in the production of thymic stromal lymphopoietin (TSLP), IL-25 (or IL-17E), and IL-33. These cytokines induce APCs, leading to activation of Th2 immune responses. In the thymus, TSLP is important for lymphocyte development, but when secreted by ECs it induces Th2 immune responses [40].

### 3.4. Th2-Inducing Cytokines Other than IL-4

TSLP is expressed in several organs, including the intestines, lungs, tonsils, and thymus, and primes DCs to enhance Th2 differentiation while inhibiting Th1-related IFNγ and IL-12 cytokines. TSLP can also activate basophils and mast cells in both humans and mice [10]. TSLP receptor-deficient mice have shown impaired Th2 responses [41], while excessive production of TSLP has been shown to increase asthmatic reactions [42]. TSLP is also known to activate TCRs by interacting with OX40 ligands, resulting in enhanced Th2 polarization [43]. Increased levels of TSLP are associated with rheumatoid arthritis and atopic diseases [44,45]. In addition to TSLP, ECs also secrete other cytokines, such as IL-25 and IL-33, which contribute to Th2 responses. IL-25, also known as IL-17E, is not only expressed in lung ECs, but also in eosinophils, basophils, mast cells, macrophages, and Th2 cells. Enhanced expression of IL-25 is reportedly associated with asthma, AD, and allergic airway inflammation in both humans and mice [46,47]. Inhalation and hypersecretion of IL-25 in airway ECs promotes Th2 inflammation in the lungs [48]. IL-25 secreted from lung ECs stimulates DCs to activate Th2 responses by upregulating expression of Jagged 1 (Notch ligand) [49]. In contrast to the role of IL-25 in directly activating CD4^+^ T cells toward the Th2 lineage, it has also been found in inhibiting Th1 and Th17 differentiation. In autoimmune inflammation, IL-25 suppresses Th17-mediated inflammation [50] and gut inflammation by inhibiting Th1 responses [51]. In humans, TSLP also activates DCs to promote Th2 immune response via IL-25 signaling [47]. The IL-25^-/-^ mouse shows susceptibility to autoimmune encephalitis and severe disease progression, including failure to eliminate the helminth, *Nippostrongylus brasiliensis*, due to poor Th2 response [51,52]. Collectively, these results suggest that cytokines secreted from ECs favor the Th2 specific immune response. IL-33 is a member of the IL-1 family and is a ligand for receptor ST2 and functions as an alarmin to activate other cell types. IL-33 inhalation potently drives production of Th2 cytokines (e.g., IL-4), resulting in activation of Th2 immune responses in both human and mouse, as shown by an increase in mucus production, IgE secretion, and eosinophilia [53]. IL-33 activates mouse DCs to stimulate the Th2 immune response during allergic airway inflammation [54,55]. IL-33 also stimulates mast cells to produce TSLP [56] and basophils to enhance the release of histamines and IL-4 in the presence of IL-3 [57], leading to activation of mast cell-basophil-driven anaphylaxis and inflammation [58]. The synergistic effect of IL-33 and TSLP has been shown to enhance the production of Th2-related chemokines and cytokines by mast cells. Collectively, IL-33, IL-25, and TSLP secreted from different cell types induce the production of Th2-related cytokines, leading to allergic inflammatory diseases, such as AD and allergic asthma [46].

## 4. Dendritic Cell Subsets and Th2 Immunity

Th2 immunity is an important defense mechanism against extracellular parasites and allergens. DCs can generate effector T-cell subsets from naïve T cells specialized for specific immune responses and are therefore considered “master regulators” of immune responses because they have the ability to initiate and control the adaptive immune response. DCs comprise a heterogeneous group of cells originating from bone-marrow hematopoietic stem cells (HSC) and early myeloid progenitors (EMP) in the blood, tissues, and lymphoid organs. They are generally classified on the basis of their locations, functions, and cell surface markers. HSCs give rise to conventional/classical DC1 (cDC1) and DC2 (cDC2), plasmacytoid DCs (pDCs), and monocyte-derived DCs (Mo-DCs) (Table 1).

EMPs give rise to monocyte-derived macrophages and long-lived resident macrophages. Development of the XCR1^+^ cDC1 subset (CD8α^+^ cDCs and CD103^+^ cDCs) is dependent on IRF8, ID2, and Batf3 transcription factors. Development of CD172^+^ CD11b^+^ cDC2 subsets is dependent on Klf4, IRF4, ID2, ZEB2, RelB, and Notch2 transcription factors. The IRF8-dependent cDC1 subset presents antigens to CD8^+^ T (CTL) cells through MHC I, mainly involved in promoting anti-viral and Th1 cell response, whereas IRF4-dependent cDC2 subsets present to CD4^+^ T cells through MHC II with the ability to favor polarization toward Th2, Th9, Th17, and Treg cells [14,59] (Figure 2). Among the IRF4^+^ cDC2 group, three subsets (CD301b^+^ DCs, PDL2^+^ DCs, and CD11b^+^ DCs) are well established to be associated with induction of the Th2 immune response in mice, as summarized by Na et al. (2016) [3]. The subsets are located mainly in the skin, lung, and spleen. Mouse CD301b^+^ DCs are a skin-resident DC subset that plays a crucial role in the Th2-mediated contact hypersensitivity response in the skin [17,59]. Mouse PDL2^+^ DCs are enriched in the lung and the draining lymph nodes (dLNs) of the skin and intestine and are involved in allergic inflammation by enhancement of Th2 immunity [60]. Neither type of cDC2 subset induces differentiation or development of Th2 immunity, but enhances Th2 responses by activating effector and memory T cells [3,59]. CD11b^+^ DCs, however, are involved in the development of Th2 responses from naïve T cells in the regional LN upon activation and CCR7-dependent migration [15,61,62]. In addition, CXCR5^+^ DCs also play an important role in inducing Th2 immunity in intestinal nematode infection [63]. Mayer et al. (2017) identified two different populations of CD11b^+^ DCs in the gut mucosal tissue, which induce Th2 response; CD11b^+^ CD103^+^ DCs in the small intestine and CD11b^+^ CD103^-^ DCs in the colon [62]. On the other hand, it is well established that in vitro-generated human Mo-DCs and mouse BMDCs can induce both CD4^+^ and CD8^+^ T cell responses through their cross-presenting capacity in nature. However, when inoculated in vivo, DC vaccines induce different polarization of CD4 vs. CD8 T cell responses in both the human and mouse, depending on the maturation conditions (hormones, cytokines, and TLR/RLR ligands) and pulsed antigens during the DC development even in the presence of same cytokines, like GM-CSF and/or IL-4 [64,65]. In addition, mouse conventional DC1 (cDC1) can also induce Th2 responses when exposed to allergens in the lung [66], and alpha-myosin-presenting cDC2 was also reported to induce Th1/Th17 in an experimental autoimmune myocarditis model [67].

## 5. DC-Mediated Th2 Immunity

**Figure 3:**
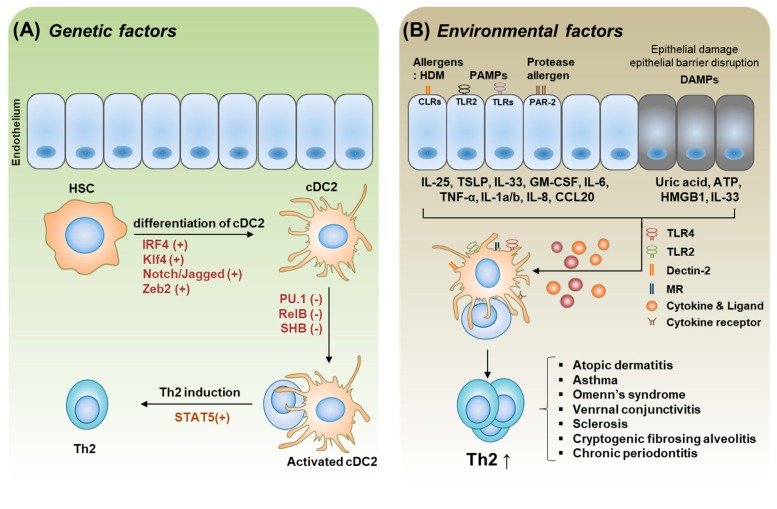
Genetic factors and environmental factors which are involved in the development of the cDC2 phenotype, eventually mediating Th2 polarization. (**A**). Genetic factors involved in each stage of cDC2 differentiation and cDC2-mediated Th2 development. Major transcription factors required for cDC2 development are marked in red and (+) as positive regulators, and (-) as negative regulators in the development of cDC2 and in controlling DC-mediated Th2 polarization. (**B**). Environmental factors affecting cDC2 priming and cDC2-mediated Th2 polarization and associated immune disorders. Allergens, PAMPs, and DAMPs, and their receptors on the ECs and DCs are indicated. Cytokines and ligand molecules secreted from damaged or primed ECs and their recognition receptors on DCs are indicated. Primed cDCs stimulate Th2 responses, occasionally leading to Th2 immune disorders. Details are described in the text.

Th2 immunity has evolved to clear multicellular pathogens. For example, because helminths are eukaryotic organisms, their physiology resembles that of humans more than it does that of unicellular organisms. Therefore, it is logical to expect that the recognition of helminths becomes more difficult for the human immune system. DCs are important APCs that can recognize such pathogens and trigger a specific Th2 immune response. IL-4 is the major cytokine required for Th2 polarization, which provides protective immunity against multicellular parasites in both humans and mice [68,69]. However, an excessive Th2 response can lead to allergic reactions. Genetic (intrinsic) and environmental (extrinsic) factors affect DC development, leading to the induction of DC-mediated Th2 responses [70]. Genetic factors comprise cDC2-specific surface molecules, certain genes (transcription factors, micro RNA, and epigenetics) required for the development of cDC2 from DC precursor cells, and other genes involved in the control of cDC2 development. Environmental factors also induce Th2 immunity by affecting cDC2 development. Helminths, HDMs, bacteria- or virus-derived PAMPs, allergens, and cytokines are all involved in DC-mediated Th2 immunity [71]. In this section, typical genetic and environmental factors affecting cDC2 development are discussed in connection with Th2 polarization and allergic inflammation (Figure 3).

## 6. Genetic Factors Required for cDC2 Development and Th2 Immunity

### 6.1. Specific Surface Features of Th2-Inducing DCs

To initiate a Th2 immune response from naïve CD4^+^ T cells, DCs require the expression of specific surface receptors to recognize and present helminth-derived antigens and allergens to Th2 cells. Chemokine receptor, CXCR5 (CXCL13) [72,73]; cytokine-receptors, TSLP receptor (CRLF2 and IL-7Rα) [74,75], IL-25R, and IL-33R (ST2); inducible costimulatory molecules ICOS (B7h) [76], OX40/OX40L [75], CD30/CD30L [77], and TIM1/TIM4 [78,79]; pattern recognition receptors (PRRs; TLR2, 3 and 4) [71]; c-type lectin receptors (Dectin-2, MGL, MR, DC-SIGN) [80,81]; RIG-l-like receptors (MDA5, LGP2) [82]; and protease-activated receptors (PAR 1–3) [83] are preferentially expressed on Th2-inducing DCs in both the human and mouse. Damage-associated molecular patterns (DAMP) receptors [84], including complement receptors (hCR1, hCR2, hCR3, mC3aR, mC5aR), prostanoid receptors (DP1, EP2, EP4, IP) [85], neuropeptide receptors (NK1, CGRPR) [86], purinergic receptors (P2X, P2Y) [87], HMGB1 receptor (RAGE) [88], and heat shock protein receptors (CD14, CD36, CD91) [89,90], are also preferentially expressed on Th2-inducing DCs. 

### 6.2. Transcription Factors (TcFs)

Among the DC subset, the cDC2 subset plays a major role in inducing Th2 immunity. Several TcFs are required for the proper development of cDC2. In this section, we summarize several TcFs, which are essential for cDC2 development leading to induction of Th2 response. Kruppel-like factor 4 (Klf4) and interferon regulatory factor 4 (IRF-4) are well-established TcFs for cDC2 development from pre-cDCs. CD11c-conditional Klf4 depleted mice showed impaired Th2 response against parasitic infection (*S. mansoni*) and HDM, probably due to the reduced populations of the cDC2 subset and IRF4^+^ pre-DCs [14]. Recently Gao et al. (2013) [13] identified that the IRF4^+^ cDC2 subset is required for Th2 immunity against a protease allergen and *N. brasiliensis* infection. STAT5 was also reported to be involved in cDC2-mediated Th2 immunity. Bell et al. found that the DC-specific deletion of STAT5 had no effect on DC development, but impaired Th2-mediated allergic responses in skin and lungs [91,92]. The proposed mechanism suggested that loss of STAT5 in DCs leads to the inability to respond to TSLP, resembling the lack of Th2 response in TSLPR^-/-^ mice [91,92]. This result indicates that the STAT5-TSLP axis in DCs is critical in promoting Th2 immunity. Notch and Notch ligands expressed in cDC2 play a crucial role in regulating Th1/Th2 polarization in both the human and mouse [93,94]. Immature DCs constitutively express Jagged-1, which induced TH2 polarization in CD4^+^ T cells while DC-specific Jagged-1 depletion inhibited Th2 polarization in humans [94]. Overexpression of Notch ligand Delta-1 in DCs exerted anti-allergic effects on Th2-mediated allergic asthma in mice [95]. This result supports a previous report that up-regulation of Notch ligands Delta-1 and Delta-4 in DCs inhibits Th2 development via the MyD88-dependent pathway [93]. Two independent studies suggest that DCs expressing TcF PU.1 play a crucial role in mediating Th1/Th2 responses. In one study, DC-specific PU.1-deficient mice induced a Th1toTh2 shift in T cell response, resulting in reduced intestinal transplant rejection in female Lewis-recipient rats due to the mixed chimerism induced by PU.1-silenced DCs [96]. In another study, the negative effect of PU.1-expressing DCs in mediating Th2 responses in mice was revealed to be due to the inhibition of GATA3 [97]. The mechanistic justification reveals PU.1 binds to a GATA3 promoter, which leads to the suppression of GATA3 expression, and high-level recruitment of the H3K4me3 heterochromatin mark at the promoter, resulting in suppression of Th2 cytokine (IL-5 and IL-13) expression. Zinc finger E-box-binding homeobox 2 (Zeb2) is an essential TcF in mediating cDC2 development from pre-cDCs. Zeb2 is expressed at the pre-pDC and pre-cDC stage and highly expressed in mature pDCs and cDC2s. CD11c-specific Zeb2-knockout mice showed decreased populations of pDCs and cDC2, but with increased population of cDC1, while, conversely, mice overexpressing Zeb2 had reduced the population of cDC1 by Zeb2-mediated targeting of Id2, a key TcF of cDC1 [98]. RelB, a member of the nuclear factor kappa-light-chain-enhancer of activated B cell (NF-kB) family is an essential TcF for DC development, maturation, and function. Adoptive transfer of RelB-deficient DCs showed the increased allergic airway inflammation with an increase in Th2-associated cytokines, IL-4, IL-5, and IL-13, in recipient mice, indicating that RelB in DCs is involved in controlling DC-mediated Th2 immune responses [99].

### 6.3. Genetic Factors Other than TcFs Involved in Th2-Inducing DC Development

Mind-bomb-1 (Mib-1), an E3 ubiquitin-protein ligase involved in regulating cell apoptosis, is a critical regulator of Notch ligands for the activation of Notch signaling, increasing gradually as precursor cells differentiate into DCs in mice. Mib-1-depleted DCs were not effective at stimulating Th2 proliferation in co-culture with T cells [100], suggesting that the Mib-1 expressed in DCs is critical for Notch-mediated Th2 differentiation. However, certain genetic factors are involved in controlling DC-mediated Th2 responses as a negative regulator. DCs deficient in expressing myeloid differentiation primary response 88 (MyD88) promoted Th2 response with a significant decrease in Th1 and Th17 cells, leading to enhanced pancreatic inflammation in both humans and mice [101]. Spontaneous mutations of the SHANK-associated RH domain-interacting protein (Sharpin or Rbckl1, Sipl1) gene in mice induce a Th2 immune response, resulting in systemic inflammation characterized by chronic progressive dermatitis [102]. Studies of the underlying mechanism showed that a Sharpin-deficiency in mice did not alter the distribution and surface phenotype of DC subtypes in the spleen, but did reduce the capacity of DCs to express pro-inflammatory Th1 cytokines and inactivated NF-kB signaling without affecting mitogen-activated protein kinase (MAPK) and TANK-binding kinase 1 signaling pathways, leading to systemic inflammation in Th2-biased response [103]. Additionally, DC-specific depletion of IL-4 receptors reportedly enhances the susceptibility to Leishmanial infection by polarizing the Th2 response [104]. Another study showed that DCs deficient in expressing IL-12 inhibit the progression of autoimmune arthritis by mediating the Th1-to-Th2 shift [105]. Gold et al. (2016) have shown that the DCs expressing SH2-containing inositol 5′-phosphatase 1 (SHIP-1) play a crucial in controlling helminthic infection by inducing a protective Th2 immune response. DC-specific SHIP1-knockout mice were highly susceptible to *Trichuris muris* infection due to insufficient priming of Th2 response with an increase in IL-12p40 production via negative regulation of the phosphoinositide 3-kinase (PI3K) pathway [106]. Webb et al. (2017) have described the role of type 1 IFN in DC-mediated Th2 polarization upon *S. mansoni* infection and HDM exposure. The DCs lacking in IFN-α receptor (ifnar1-/-) are unable to initiate Th2 response by impairing the optimal DC phenotype, suggesting its critical role in DC-mediated Th2 immunity [107].

### 6.4. Src Homology 2 Domain-Containing Adaptor Protein B (SHB)

**Figure 4:**
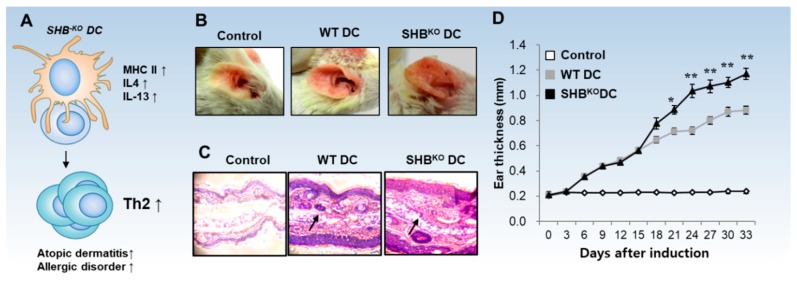
SHB-depleted DCs induce severe symptoms of AD in mice. (**A**) SHB^KO^ DCs show typical cDC2 phenotypes, which induce Th2 inflammatory disorders. (**B**) In the mouse AD model, the mice subcutaneously injected with antigen-primed SHB-depleted DCs showed severe AD symptoms as compared with the control mice injected with SHB-normal DCs. (**C**) Histopathological analysis of AD after inoculation with normal and SHB^KO^ DCs. Arrow indicates the infiltrated immune cells. (**D**) Graphical representation of ear thickness in mice after inoculation with normal and SHB^KO^ DCs. Figure source Ahmed et al. (2017) [15].

SHB is widely expressed in immune cells and acts as an important regulator in immune cells. SHB is mainly involved in mediating signals from activated tyrosine kinase receptors as well as the TCR in both humans and mice [108,109,110]. In activated T cells, SHB associates with the *ζ*-chain of TCR and promotes the phosphorylation and activation of central TCR signaling components [110]. SHB-deficient CD4^+^ T cells were hyper-proliferative and polarized toward a Th2 profile under in vitro stimulation [111]. SHB-knockout mice developed more symptoms of AD, with increased levels of IL-4, IL-5, and IgE, together with epidermal hyperplasia [111]. These data suggest that SHB in T cells plays an important role in controlling Th2-driven inflammation and allergic responses. Recently, Ahmed et al. (2017) reported that SHB is highly expressed in mouse splenic DCs and in vitro-generated BMDCs, and SHB-deficient BMDCs induce Th2 polarization in T/DC co-cultures [15]. When SHB-deficient DCs were inoculated into mice with atopic dermatitis, mice developed more severe disease symptoms [15]. SHB expression in DCs was found to be regulated by p-38-MAPK signaling-mediated Foxa2 expression and activation [15]. Inhibiting the MAPK pathway using a specific inhibitor (SB203580) significantly down-regulated SHB expression and Foxa2 phosphorylation in DCs, and Foxa2 depletion also directly inhibited SHB expression in DCs. SHB-deficient DCs showed typical cDC2 phenotypes: Enhanced expression of MHC-II and costimulatory molecules with no change in MHC-I expression, elevated levels of Th2 cytokines (IL-4 and IL-13) with no increase in IFN-γ level, and a decrease in CD4^+^CD25^+^Foxp3^+^ Treg population in OT-II T-cell/DC co-cultures. The severity and rate of development of AD increased in BALB/c mice inoculated with SHB-deficient DCs compared with mice inoculated with normal DCs (Figure 4). Collectively, these studies suggest that SHB expression in DCs is crucial for controlling DC-mediated pathologic Th2 inflammation and allergic disorders.

### 6.5. Epigenetic Factors

Epigenetic factors also contribute to allergic reactions via DC-mediated Th2 response. Alexey et al. (2010) [112] found that the DNA methylation pattern in DCs causes allergic reactions by enhancing Th2 cells’ response in mice. They found that a neonate from an asthmatic mother is more susceptible to allergic responses compared with newborns from a control mother. When they transferred the DCs from the newborn of the asthmatic mother into normal recipient mice, airway responsiveness upon ovalbumin challenge increased significantly. Epigenetic analysis of the neonates born to an asthmatic mother revealed high levels of DNA methylation from birth. This indicates that, even with the identical genomic constitution, DNA methylation in DCs may cause an allergic response by enhancing Th2 response. Another study showed that methyl-CpG-binding protein (Mbd2) epigenetically controls DC-mediated Th2 immunity in mice [113], revealing that DCs’ lack of Mbd2 expression could not induce appropriate Th2 response against helminthic (*S. mansoni*) infection due to the impairment of Mbd2-mediated H3K9/K14 acetylation, leading to reduced expression of Th2-inducing genes in DCs. 

### 6.6. MicroRNA

MicroRNAs have been associated with several allergic inflammatory disorders, including asthma, eosinophilic esophagitis, and allergic rhinitis as well as AD. However, most studies have been performed in association with T-cell development. Recently, Zech et al. (2015) reported that microRNA-155 (miR-155)-deficient DCs showed limited Th2 priming capacity and failed to induce airway inflammation in allergen-exposed mice due to impairment of the miR-155-mediated purinergic type 2 receptor (P2R) signaling activation, resulting in inhibition of DC chemotaxis and IL-1beta secretion upon stimulation [114]. This means that miR-155 is essential for DC-mediated Th2 inflammatory response.

## 7. Environmental Factors

Apart from genetic factors, several environmental or extrinsic factors facilitate the DC-mediated Th2 immune responses. Most environmental factors or variables drive the immune system to determine the fate of DC-mediated T cell responses, i.e., whether Th1 or Th2 immune responses are elicited in the body. In in vitro cultures, the antigen dose and the DC/T-cell ratio in the same culture condition also affect the DC-mediated Th2 response. For example, bone marrow-derived mouse myeloid DCs cultured in the presence of high doses of antigen induce Th1 cell development, whereas low antigen doses induce Th2 cell development [115]. Human Mo-DC cultures with naïve T cells at a low ratio (1:300) induce Th2 cells, whereas a high ratio (1:4) favors mixed Th1 and Th2 cell development [116]. In addition, DC maturation conditions also affect Th1/Th2 polarization [117]. Following are some of the environmental factors discussed.

### 7.1. Allergens

Allergens, such as pollens and HDMs, can lead to the induction of Th2 immune responses. Exposure of innate immune cells, i.e., eosinophils, basophils, and inflammatory DCs (iDCs), in the mediastinal draining lymph nodes (dLNs) to HDMs activates the TLR4/MyD88-dependent pathway, which recruits the IL-4 competent Th2 immune response. FcεRI^+^ iDCs present antigens to T cells after exposure to HDMs and induce Th2 immune responses that lead to features of asthma in mice [3]. A study in mice has shown that following HDM inhalation, blood DCs recognize the HDM antigens via the C-type lectin receptor (CLR) dectin-2, which causes the production of cysteinyl leukotrienes, hence leading to the pro-allergic responses [118,119]. Another CLR, the mannose receptor (MR) in DCs, mediates the uptake of HDM allergens and induces Th2 polarization through upregulation of indoleamine 2, 3-dioxygenase activity [120]. The role of DCs has been well-established in association with the mechanism of food allergy. Cow’s milk causes allergic reactions in healthy mice when DCs are adoptively transferred from allergic mice [121]. This DC-induced allergy in recipient mice was characterized by the presence of cow’s milk-specific immunoglobulins (i.e., IgE and IgG) and by the resistance to apoptosis by milk-specific Th2 cells. This apoptosis-resistance feature of Th2 immune responses has been attributed to the donor-specific DC subsets [122]. In a cholera toxin (CT)-induced food allergy model, oral administration of peanut extract and CT induced a shift of DC subsets toward more cDC2-type (CD11c^+^CD11b^+^) than cDC1 types (CD11c^+^CD103^+^) in the mucosa [123], which mediated CT-induced Th2-skewing via up-regulated OX40L in DCs [124]. In addition, peanut allergen and HDM glycoprotein Ara h1 were observed to bind to a CLR, DC-specific, intercellular adhesion molecule 3-grabbing nonintegrin (DC-SIGN), and stimulate human MoDCs to induce Th2 immunity, but deglycosylated Ara h1 did not show a Th2-skewing effect [122]. It means that allergen-bound carbohydrate structures may act as a Th2-skewing adjuvant.

### 7.2. PAMPs and DAMPs

PAMP molecules in invading microbes are recognized by innate immune cells, such as DCs, via specific pattern recognition receptors (PRRs) and activate the immune system [3]. Studies have shown that the activation of DCs in response to pathogens requires the presence of a PAMP molecule to induce Th1 or Th2 polarization in both humans and mice [125]. For example, the trematode, *S. mansoni*, lays soluble eggs in mice that contain an antigen with a glycosylated T2 ribonuclease, termed as omega-1, which activate DCs to induce Th2 immune responses [126]. Omega-1 drives DC-mediated Th2 polarization by suppressing protein synthesis in DCs after internalization via the mannose receptor [127]. Similarly, lipopolysaccharides (LPS) from gram-negative bacteria are reportedly involved in inducing both Th1 as well as Th2 immune responses based on quantity or dose. A high dose of LPS induces Th1 while a low dose activates the TLR4-dependent pathway in DCs to induce Th2 immune responses [128]. Apart from the PAMPs, certain PRRs, such as TLRs, are also involved in driving Th2 polarization by DCs. Pam-3-Cys, a TLR2 ligand (TLR2L), stimulates DCs via TLR2, leading to DC-mediated Th2 immune responses via the ERK-cFos pathway. Following ERK activation by Pam-3-Cys, the TcF cFOS is phosphorylated, which inhibits IL-12p70 expression, leading to the induction of Th2 responses in mice [129]. A nematode glycoprotein excretory-secretory-62 induces DC-mediated Th2 skewing via TLR4 in mice [130,131]. Similarly, TLR4-mediated Th2 priming via DCs is also shown by the LNFPIII glycol-conjugate in *Schistosoma*’s soluble egg antigen [132]. Tissue injury or damage provokes the release of the DAMPs, which are known to be potent Th2 inducers [42]. Adjuvant alum and high-mobility group nucleosome binding protein 1 (HMGN1) are well-established DAMP molecules known to induce DC-dependent Th2 polarization [43].

## 8. Diseases Associated with DC-Mediated Th2 Immunity

More than one billion people worldwide are suffering from parasitic infections, helminths, and allergic disorders, such as asthma, allergic rhinitis, food allergies, and eczema [133,134,135]. Common features of these inflammatory diseases are so-called an allergic or “type 2” immune disorder [136]. A number of animal models have been developed to study the pathogenic mechanism of these allergic diseases and obtain better insight into the orchestration of immune-related pathological mechanisms. Murine models are most frequently used to study the development of allergic sensitization, elicitation, and the potential of immunotherapeutic interventions. However, the results obtained from an animal model must be interpreted with caution, as they may not be applicable to human immune diseases. Diseases associated with Th2 dysfunction in humans include Omenn syndrome [137], asthma [95,138,139], AD [140,141,142], progressive systemic sclerosis [143], cryptogenic fibrosing alveolitis [144], chronic periodontitis [145], progression to AIDS in HIV infection [146], and tumor progression in both human and mouse models [147]. Among these, typical Th2-mediated immune disorders are discussed in this section.

### 8.1. Parasitic Infections

Parasitic infections, especially helminthic infections, occur in almost one-quarter of the world’s population [148]. These helminths elicit the innate immune response system to produce Th2 immune responses against invading parasites. These Th2 cells then secrete their respective cytokines, i.e., IL-4, IL-5, and IL-13. Alongside Th2, these cytokines help promote IgE production by B cells as well as recruit eosinophils and activated macrophages [69]. It has been shown that by depleting the CD11c^+^ DCs, the Th2 immune responses are interrupted in infections of the helminths, *Heligmosomoides polygyrus* and *S. mansoni* [149,150,151]. Recent studies have shown that different DC subsets are implicated in Th2 immune responses. Dermal CD301b^+^ DCs are involved in Th2 immune responses against *N. brasiliensis* in a mouse model [13,59]. Meanwhile, *H. polygyrus* and *N. brasiliensis*, but not *T. muris*, produce excretory-secretory products and thereby suppress the production of IL-12p40 from DCs in mice [152]. This causes induction of TSLP, which is crucial to induce Th2 immune responses, especially against *T. muris* in a mouse model [153]. Therefore, the impairment of DC in parasitic infections suppresses the Th2 immunity and rather favors the Th1 immune response in the adaptive immune system. The linking of innate and adaptive immune branches is often associated with the different types of TLRs [154]. When mice were infected by parasites and stimulated via TLR-mediated signaling, DCs secrete pro-inflammatory cytokines and thus upregulate the expression of costimulatory molecules [155]. However, endotoxins, especially LPS, can activate the MyD88-independent pathway. MyD88 is a TLR-associated adaptor protein that is crucial for TLR-mediated cytokine production. However, a recent study has shown that MyD88^-/-^ DCs can maintain the expression of costimulatory molecules in response to the endotoxin, and elicit Th2 immune responses in mice [156]. It means that endotoxin can induce DC-mediated Th2 immune disorder via the MyD88-independent pathway 

### 8.2. Asthma (AS)

cDC2s play a vital role in generating Th2-mediated immune responses in asthma, including HDM-mediated asthma in a mouse model [157,158,159]. The cDC2s induce both Th2 and Th17 differentiation in HDM asthma. Various innate receptors are found on the surfaces of this cDC2 subset that recognize HDM. Dectin-2 is an innate receptor on the surface of cDC2 subset, which recognizes HDM antigens and helps DCs to uptake allergen in mice [118,160]. IRF4-depleted mice show cDC2 deficiency, leading to a reduction of Th2 immune responses [13,161]. cDC2s also express OX40L, a TNF family member, which is important for the induction of Th2-mediated asthma. HDM-induced IL-33 production in neonatal mice inhibits expression of IL-12p35 and induces expression of OX40L in cDC2s, thereby inducing cDC2-mediated Th2 allergic disorder [162,163].

### 8.3. Atopic Dermatitis (AD)

AD, also known as eczema, is one of the most common forms of skin ailments. It is characterized by IgE-mediated hypersensitivity to environmental or food allergens and by dry and inflamed skin in humans [164]. In patients with AD, epidermal DCs carry FcεRI on their surfaces, which is the high-affinity receptor for IgE [165,166,167]. AD is characterized by the presence of two major cell types, Langerhans cells (LCs) and inflammatory epidermal DCs. Both of the cell types highly express the FcεRI receptor in humans [168]. LCs normally reside in the skin, but iDCs localize only where inflammation occurs. These cells take up the allergen and present it to either Th1 or Th2 cells depending on the cell types. Human LCs present to Th2 cells while iDCs present to Th1 cells [169,170]. 

### 8.4. Allergic Rhinitis (AR)

Also known as hay fever, AR is a nasal inflammation that occurs when the body is exposed to an airborne allergen. It manifests as nasal discharge, sneezing, and ocular discharge with redness and swelling of the eyes in human [171]. In AR, Th2 cytokines are induced in CD4^+^ T cells by mDCs (myeloid DCs) expressing lower levels of the ICOS ligand, which is a costimulatory molecule important for DC-T-cell crosstalk. Although ICOS is linked to Th1 responses, its expression is significantly reduced in patients with allergic rhinitis [172], whereas OX40L expression is significantly increased and has a sentinel role in promoting Th2 polarization of CD4^+^naïve T cells within the LNs. Therefore, it has been suggested that the OX40L and TSLP may be a therapeutic target for AR patients [173,174,175].

## 9. Treatment for Th2-Polarized Immune Disorder

Parasitic infections or allergen invasion stimulate Th2 immunity by expressing Th2 associated cytokines. However, prolonged secretion of Th2 cytokines occasionally leads to adverse allergic reactions in humans, characterized by high serum levels of IgE in allergic disorders, such as AD, asthma, rhinitis, and hay fevers. Thus, the secretion of Th2 cytokines needs to be tightly controlled. Although a detailed therapeutic mechanism is beyond the scope of this review, we briefly discuss the current therapeutic approaches, which are focused on the Th1/Th2 immune balancing from Th2 skewing immunity. The idea behind developing and designing any drug is mainly based on three approaches: First, by blocking the key factor required for secretion of Th2 cytokines; second, by targeting the important signals required for differentiation and survival of Th2 immune cells; and third, by activating Th1 immunity to restore the Th1/Th2 immune balance. For example, eosinophils, a well-known myeloid lineage, rapidly infiltrate into the regions of inhaled allergens or parasitic infections, leading to the conscription of other immune cells, such as DCs, mast cells, basophils, and NKT cells, to enforce Th2 responses. Targeting these intermediate pathways could be the best approach to designing Th2 drugs. In this section, we reviewed the current therapeutic approaches for the treatment of Th2 immune disorders in the aspect of pharmacology, biologics, and molecular targets [176] (Table 2).

### 9.1. Pharmacological

Tacrolimus (FK506) and Cyclosporine-A are the well-known immunosuppressive drugs, which have been used successfully in the treatment of AD, and organ transplantation to prevent graft-versus-host diseases by targeting NFAT and AP1 proteins, leading to inhibition of calcineurin and IL-4 production in both humans and treated animals [215,216]. Parthenolide acts as an anti-inflammatory drug used for the treatment of allergic disease by suppressing IL-4 in both humans and mice [179,180]. Aspirin has been successfully used in the treatment of allergic diseases, like childhood asthma, which inhibits STAT6 activation via the IL-4 and IL-13 signaling pathway [181]. Vitamin E has been used for the treatment of AD patients because it is a potent antioxidant, with the ability to decrease the serum IgE level by blocking IL-4 secretion through interfering with the NF-κB and AP1 binding to P1 and PRE-I/P4 sites on the IL-4 promoter in both mouse and human cells [177,178].

### 9.2. Biological (Anti-Interleukins)

Several anti-interleukins are being used successfully in the treatment of Th2 mediated immune disorders (Table 1). In addition to biologics, genetically engineered DCs expressing IL-4 have been shown in controlling arthritis in a mouse collagen-induced arthritis model [217]. Mouse DCs especially myeloid DCs and epidermal LCs expressing CCL17 and CCL22, a chemoattractant of Th2 cells, have been reported to have capacity in controlling AD by blocking the IL-4/STAT-6 signaling pathway [218]. The details of the DC-mediated vaccination and immunotherapeutic approach in both humans and mice for Th2 skewing allergic disorders are well described in the following references [176,219,220,221,222,223,224,225].

### 9.3. Molecular (microRNA)

MicroRNAs are recently counted on the list of treatment categories for Th2-mediated immune disorders, which are summarized briefly in Table 1. It was reported that miR-135a controls AR by regulating Th1/Th2 immune balancing in the murine model [212]. MicroRNA-155 regulates various steps of DC-associated Th2 responses by targeting PU.1 transcription factor in controlling allergic airway inflammation in mice [209]. Additionally, miR-155 and miR-146a regulate ‘S1pr1’ a bioactive lipid compound in controlling inflammation [208]. MiR-106b has been shown in controlling allergic inflammation by negatively regulating BMDC-mediated Th2 polarization in mice through targeting early growth response gene-2 ‘Egr2’ [213]. Qui et al. (2017) have shown that miR-371, miR-138, miR-544, miR-145, and miR-214 can modulate the Th1/Th2 balance in a mouse asthma model through the combinatorial regulation of Runx3 [214].

### 9.4. Hygiene Hypothesis

In addition to biological and pharmacological treatment, hygiene hypothesis theory recently gained more attention towards preventing allergic disease [226,227,228,229]. Bart et al. (2017) described that the ECs as an outer barrier are constantly influenced by environmental factors. The danger signals and metabolites derived from the microbiome also trigger the immune response and thus it is important to rheostats immunoregulation. Thus, preventing strategies could be implemented to control the allergenic responses. Parasitic infection especially helminth has also been shown to control human allergic disease and autoimmune disorders [230].

## 10. Conclusions and Future Directions

As professional antigen presenting cells, DCs play a major role in controlling and maintaining the immune homeostasis in the body by means of antigen recognition and presentation to the naïve T cells to induce specific immune responses against harmful antigens. This review described the current understandings of the Th2 immunity induced by specific DC subsets, especially the cDC2s, and their roles in determining the specific pathways that drive and determine the Th1 versus Th2 immune responses. Th2-specific antigens or allergens readily recognized and presented by DCs eventually lead to the induction of the Th2 immune responses, resulting in the clearance of the antigen or may cause Th2-skewing chronic inflammatory disorders. In this review, we have discussed the DC-mediated Th2 polarization in connection with intrinsic, i.e., genetic factors, such as certain gene-related mutations or certain DC-specific genetic aberrations, and extrinsic, i.e., environmental factors, such as allergens, PAMPs, DAMPs, TLRs etc. The genetic factors required for the cDC2 development are essential for the induction of the DC-mediated Th2 immunity while specific environmental factors, such as the allergens or the extracellular parasites, that drive DCs to elicit Th2 polarization in the body. The detailed mechanisms underlying the induction of DC-mediated Th2 immunity to each antigen remain to be further elucidated, which will provide a better understanding of the DC-mediated Th2 immunity, eventually making progress in the field of drug development and therapeutic interventions for Th2-biased chronic inflammatory disorders.

## Figures and Tables

**Table 1:** Dendritic cell subtypes in the human and mouse.

	Human		Mouse	
Classification	Main Surface Marker	Major TcFs	Main Surface Marker	Major TcFs
**pDC**	**CD123(IL-3R)**, CD45R, CD303(CLEC4C) CD304(BDCA-34), CD85κ(ILT3), CD85g(ILT7), FCεR1, BTLA1, CD300A	TCF4/E2-E, IRF4/7/8, Zeb2	**CD317**, **Siglec-H**, CD45R, CD45RA	TCF4/E2-E, IRF8
**cDC1**	**CD141(BDCA-3)**, CD13, CD33, CLEC9A, CADM1(NECL2), BTLA, XCR1	BATF3, IRF8, ID2	**CD8α**, **CD103**, DEC205, XCR1	BATF3, IRF8, ID2, BCL6, PU.1, E4BP4
**cDC2**	**CD1c**, CD2, FCεR1, CD11b, CD11c, CD1a	ID2, IRF4, KLF4, Notch2, RBPJ	**CD11b**, SIRPα, CD301b, PD-L2	IRF4, KLF4, Notch2, ID2, Zeb2, RelB, SHB, STAT5
**Mo-DC**	**CD1a**, **CD1c**, CD11b, CD14, CD16, CD19, CD20, FCεRIα	MAFB, KLF4	**CD11b**, CD209, Ly6C, Ly6G, CD64, F4/80	IRF4, Zbtb46

**Table 2:** Approaches for treatment of Th2-mediated immune disorders.

Drug	Target	Disorder	References
**Pharmacological (Inhibitors)**			
CsA & FK506	NFAT, AP1	AD	[137,138]
Vitamin E	NF-κB, AP1	AD	[177,178]
Parthenolide	IL-4	Allergy	[179,180]
Aspirin	STAT6	Allergy, AS	[181]
**Biologics (IL-4, 5, 13 directed therapies)**			
Omalizumab	Anti-IgE	AD, AS, AR, CSU, EE	[182,183,184,185,186]
Dupilumab	Anti-IL-4/IL-13	AD, AS, NP	[187,188]
Lebrikizumab	Anti-IL-4/IL-13	AD	[189,190,191]
Tralokinumab	Anti-IL-13	AD	[192,193]
Pascolizumab	Anti-IL-4	AS	[194]
Pitrakinra	Anti-IL-4/IL-13	AD, AS	[195]
Mepolizumab	Anti-IL-5	AD	[196,197,198,199]
Reslizumab	Anti-IL-5	AS	[200]
Benralizumab	Anti-IL-5	AS	[201]
Anrukinzumab	Anti-IL-13	AS, ulcerative colitis	[202]
Ligelizumab	Anti-IgE	AD, AS	[203]
Nemolizumab	Anti-IL-31	AD	[204]
Ustekinumab	Anti-IL-12/23	Psoriasis, AD	[205,206]
Fezakinumab	Anti-IL-22	AD	[207]
**TSLP directed therapy**			
Tezepelumab	Anti-TSLP	AD	[207]
**Molecular Targets (microRNA)**			
miR-155	c-Maf (IL-4 promoter)	AD, Allergy	[208,209]
miR-126	Repress IL-4, 5, 13	Allergy	[210]
miR-133b	Nlrp3	AR	[211]
miR-135a	GATA3	AR	[212]
miR-106b	Egr2	Allergy	[213]
miR-138,371,544,145,214	Runx3	AS	[214]

## Abbreviations

AD	atopic dermatitis
APCs	antigen presenting cells
AR	allergic rhinitis
AS	asthma
BMDCs	bone marrow-derived DCs
cDCs	conventional/classical dendritic cells
CSU	chronic spontaneous urticarial
CIA	collagen-induced arthritis
CLRs	c-type lectin receptors
CRn	chronic rhinosinusitis
CXCL13	chemokine receptor CXCR5
DAMP	damage associated molecular pattern
DCs	dendritic cells
DC-SIGN	dendritic cell–specific intercellular adhesion molecule 3–grabbing nonintegrin
DTR	diphtheria toxin receptor
EE	eosinophilic esophagitis
ECs	epithelial cells
EMP	early myeloid progenitors
ER	endoplasmic reticulum
HDM	house dust mite
Hu-mAb	humanized monoclonal antibody
HSC	hematopoietic stem cells
IDO	indoleamine 2, 3 - dioxygenase
IL	interleukin
ILCs	innate lymphoid cells
IFN-γ	interferon-gamma
IPF	idiopathic pulmonary fibrosis
IRF4	interferon regulatory factor
KHIDI	Korea health industry development institute
Klf4	kruppel-like factor 4
Lfα	lymphotoxin α
MC	mast cell
MHC	major histocompatablility complex
miR	microRNA
MM	macrophage mannose receptor
Mo-DCs	monocyte derived dendritic cells
MyD88	myeloid differentiation primary response 88
Mib-1	mind-bomb-1
MIIC	MHC II-containing Compartment
NF-kB	nuclear factor kappa B
NKT	natural killer T cells
NLRP3	NLR family pyrin domain containing 3
NP	nasal polyposis
NRF	national research foundation
PAMP	pathogen-associated molecular pattern
PAR 1-3	protease activated receptors
pDC	plasmacytoid dendritic cells
PE	peripheral eosinophilia
PRRs	pattern recognition receptors
P2R	purinergic type 2 receptor
Sharpin	SHANK-associated RH domain-interacting protein
SHB	SH2 domain-containing adaptor protein B
STAT	signal transducer and activator of transcription
TAP	transporter associated with antigen processing
TcF	transcription factors
TCR	t-cell receptor
Th2	t helper type 2
TLR	toll-like receptor
TNF-β	tumor necrosis factor-beta
Treg	regulatory T cells
TSLP	thymic stromal lymphopoietin
UC	ulcerative colitis
Zeb2	zinc finger E-box-binding homeobox 2
